# The *p* factor of psychopathology and personality in middle childhood: genetic and gestational risk factors

**DOI:** 10.1017/S0033291723000077

**Published:** 2023-07

**Authors:** Line C. Gjerde, Espen Moen Eilertsen, Tom A. McAdams, Rosa Cheesman, Terrie E. Moffitt, Avshalom Caspi, Thalia C. Eley, Espen Røysamb, Tom H. Rosenström, Eivind Ystrom

**Affiliations:** 1 Department of Mental Disorders, Norwegian Institute of Public Health, Oslo, Norway; 2 Promenta Research Center, University of Oslo, Oslo, Norway; 3 Centre for Fertility and Health, Norwegian Institute of Public Health, Oslo, Norway; 4 Social, Genetic & Developmental Psychiatry Centre, Institute of Psychiatry, Psychology & Neuroscience, King's College, London, UK; 5 Department of Psychology and Neuroscience, Duke University, Durham, USA; 6 Department of Child Development, Norwegian Institute of Public Health, Oslo, Norway; 7 Department of Psychology and Logopedics, Faculty of Medicine, University of Helsinki, Helsinki, Finland; 8 School of Pharmacy, University of Oslo, Oslo, Norway

**Keywords:** p factor, MoBa, behavior genetics, factor analysis, psychiatric comorbidity, internalizing and externalizing behavior problems

## Abstract

**Background:**

A joint, hierarchical structure of psychopathology and personality has been reported in adults but should also be investigated at earlier ages, as psychopathology often develops before adulthood. Here, we investigate the joint factor structure of psychopathology and personality in eight-year-old children, estimate factor heritability and explore external validity through associations with established developmental risk factors.

**Methods:**

Phenotypic and biometric exploratory factor analyses with bifactor rotation on genetically informative data from the Norwegian Mother, Father, and Child Cohort (MoBa) study. The analytic sub-sample comprised 10 739 children (49% girls). Mothers reported their children's symptoms of depression (Short Moods and Feelings Questionnaire), anxiety (Screen for Anxiety Related Disorders), attention-deficit/hyperactivity disorder inattention and hyperactivity, oppositional-defiant disorder, conduct disorder (Parent/Teacher Rating Scale for Disruptive Behavior Disorders), and Big Five personality (short Hierarchical Personality Inventory for Children). Developmental risk factors (early gestational age and being small for gestational age) were collected from the Medical Birth Registry.

**Results:**

Goodness-of-fit indices favored a *p* factor model with three residual latent factors interpreted as negative affectivity, positive affectivity, and antagonism, whereas psychometric indices favored a one-factor model. ADE solutions fitted best, and regression analyses indicated a negative association between gestational age and the *p* factor, for both the one- and four-factor solutions.

**Conclusion:**

Correlations between normative and pathological traits in middle childhood mostly reflect one heritable and psychometrically interpretable *p* factor, although optimal fit to data required less interpretable residual latent factors. The association between the *p* factor and low gestational age warrants further study of early developmental mechanisms.

## Introduction

In psychopathology, comorbidity is common. Around half of people who meet diagnostic criteria for one disorder simultaneously meet criteria for other disorders (Newman, Moffitt, Caspi, & Silva, 1998). The need to understand comorbidity in mental health has inspired research on the structure of psychopathology using factor-analytic methods. A two-factor model, encompassing an internalizing factor characterized by negative mood states and behavioral inhibition, and an externalizing factor, characterized by behavioral disinhibition explain cross-disorder correlations well in samples of both children (Achenbach, 1992) and adults (Krueger, 1999). However, the extensive cross-correlation between the internalizing and externalizing spectra themselves (Cosgrove et al., 2011; Lahey et al., 2008) has made the notion of a continuous general factor of psychopathology (often referred to as p; Caspi et al., 2014) increasingly popular in summarizing and explaining liability to psychopathology (although other approaches to comorbidity exist – such as severity and directionality assessments [Marceau & Neiderhiser, 2022)].

Cross-correlations and one overarching *p* factor of psychopathology suggest that categorical nosologies of psychopathology fall short of capturing the complexity in psychopathology. As a response, the Hierarchical Taxonomy of Psychopathology (HiTOP; Kotov et al., 2017) works toward an alternative nosology based on a dimensional model of psychopathology. Following this work, there is a growing consensus about the importance of personality (characteristic ways of thinking, feeling, and behaving) particularly in the form of the Big Five framework (Goldberg, 1990; McCrae & Costa, 1987), for psychopathology (Widiger et al., 2019). First, the HiTOP superspectra align closely with the Big Five personality dimensions (neuroticism, extraversion, openness, agreeableness, and conscientiousness; Kotov et al., 2017; Widiger et al., 2019), and *p* factors of personality and psychopathology correlate strongly (McCabe, Oltmanns, & Widiger, 2022). Second, personality contributes substantially to different life outcomes (Ozer & Benet-Martínez, 2006) including common mental disorders (e.g. Kotov, Gamez, Schmidt, & Watson, 2010). Third, the HiTOP postulates inclusion of personality traits assessment to predict future psychopathology (Widiger et al., 2019), recently demonstrated by Waszczuk et al., who found that personality traits better predicted future psychopathology than previous psychiatric diagnoses (Waszczuk et al., 2021).

We and others have previously shown that correlations between personality and psychopathology can be rotated to a general behavioral risk factor (McCabe et al., 2022; Rosenström et al., 2018). However, attempts to investigate the joint factorial structure of psychopathology and personality have only been preliminary in childhood (Shields, Giljen, España, & Tackett, 2021). The *p* factor in childhood is poorly understood (Levin-Aspenson, Watson, Clark, & Zimmerman, 2020). Some find that *p* is predominantly linked with internalizing symptoms (Lahey, Van Hulle, Singh, Waldman, & Rathouz, 2011; Tackett et al., 2013), others with externalizing symptoms and inattention (Moore et al., 2020; Olino et al., 2018), particularly when personality is included (Slobodskaya, 2014). Mixed findings could be due to variations in content sampling and the age span included (Levin-Aspenson et al., 2020). In this study, we focus our investigation on middle childhood (age 8 years), a period marked by dramatic changes in self-regulation, executive functions, and mentalization (DelGiudice, 2018). As personality traits are more easily identifiable than psychopathology in prepubertal children, the present research could identify potential personality trait antecedents of psychopathology that may ultimately be intervened on.

Critiques of the *p* factor put forward that the *p* factor is only descriptive, and not more than the sum of its parts (Fried, Greene, & Eaton, 2021). In the present study, we seek to convey that the *p* factor is a useful construct in understanding etiology, thus moving beyond mere description, if it (1) captures early genetic and environmental risk for psychopathology in childhood, (2) demonstrates basic psychometric properties (Bonifay, Lane, & Reise, 2016), and (3) relates to putative early risk factors for psychopathology, in line with the nomological network thinking for construct validity (Cronbach & Meehl, 1955).

The *p* factor and personality traits are heritable (Allegrini et al., 2020; Waldman, Poore, van Hulle, Rathouz, & Lahey, 2016). There is also evidence of genetic correlations between psychopathology and personality (Blonigen, Hicks, Krueger, Patrick, & Iacono, 2005; Bouchard & McGue, 2003; Czajkowski et al., 2018). However, the heritability of a common childhood *p* factor, with personality included, has not been estimated.

Gestational age and being small for gestational age (SGA) are associated with poorer functioning in several domains (Gluckman & Hanson, 2006; Wolke, Johnson, & Mendonça, 2019). For instance, children born preterm or with low birth weight have significantly more internalizing and externalizing problems in childhood, adolescence, and young adulthood (Hack et al., 2004; Laerum et al., 2019; Mathewson et al., 2017). SGA has been found to be associated with a *p* factor in adults when familial confounding is controlled for (Pettersson, Larsson, D'Onofrio, Almqvist, & Lichtenstein, 2019). One possible pathway from gestational risk factors to later psychopathology is through compromised brain development, for instance due to a lack of oxygen and nutrients during a critical period (Kapellou et al., 2006; Walhovd et al., 2012), another is through social factors such as parenting (Wolke et al., 2019).

Using a large, population-based birth cohort of eight-year-old children with measures on a broad range of psychopathology traits as well as on Big Five personality we aim to (1) explore the joint, hierarchical structure of psychopathology and personality traits in middle childhood; (2) estimate genetic and environmental contributions to the obtained latent variables; and (3) investigate associations between putative early risk factors (gestational age and SGA) and the obtained latent variables.

## Methods

### Sample

This study is part of the Norwegian Mother, Father and Child Cohort Study (MoBa), conducted by the Norwegian Institute of Public Health. MoBa is a prospective, ongoing pregnancy cohort study (Magnus et al., 2016). Participants were recruited from 1999 to 2008 at a routine ultrasound examination offered to all pregnant women in Norway at gestational week ≈18. The total sample includes >114 500 children, >95 000 mothers and >75 000 fathers. In total, 41% of eligible women participated. The current study is based on the genetically informative subproject called the Intergenerational Transmission of Risk, where the wider kinship (e.g. twins, siblings, cousins) between participants in both the parent and the child generation has been identified (eAppendix 1). The present study consisted of 10 739 children (49% girls) with a relative also participating in the MoBa study. In the study sample there were 117 monozygotic twin relations, 4261 dizygotic twin and sibling relations, 108 half-sibling relations, 2354 cousin relations and 96 half-cousin relations. The additive genetic correlations between these types of relatives are 1.0, 0.5, 0.25, 0.125 and 0.0625, respectively. Non-additive genetic correlations are 1.0 for monozygotic twins, 0.25 for dizygotic twins and full siblings, and 0.00 for the rest of the relations. Among the relatives, there were 4420 shared-mother relations (necessary to model shared environmental influences as discussed in the biometric modeling procedure below).

Version 11 of the quality-assured MoBa data files were used, released in 2018. Written informed consent was obtained from all participants upon recruitment. The establishment and data collection in MoBa was previously based on a license from the Norwegian Data protection agency and approval from The Regional Committee for Medical Research Ethics, and is now based on regulations related to the Norwegian Health Registry Act. The current study was approved by The Regional Committee for Medical Research Ethics.

### Measures

Depressive symptoms were reported by mothers using the 13-item Short Moods and Feelings Questionnaire ( Angold et al., 1995). Anxiety symptoms were reported by mothers using the five-item version of the Screen for Anxiety Related Disorders (SCARED; Birmaher et al., 1997). Attention-deficit/hyperactivity disorder (ADHD), oppositional-defiant disorder (ODD), and conduct disorder (CD) symptoms were reported by mothers using the Parent/Teacher Rating Scale for Disruptive Behavior Disorders (RS-DBD; Silva et al., 2005). We analyzed inattention and hyperactivity in ADHD separately due to recent evidence on differential etiologies (Gustavson et al., 2021). Nine items were each used to measure ADHD inattention and hyperactivity, and eight items to measure ODD and CD, respectively. We created sum scores of the scale items for each of the six traits.

Big Five personality (neuroticism, extraversion, imagination, conscientiousness and benevolence/agreeableness) was reported by mothers using the short Hierarchical Personality Inventory for Children (HiPIC-30; Vollrath, Hampson, & Torgersen, 2016). Each personality trait was constructed using the sum of six items. More information on the psychopathology and personality scales (e.g. items and response categories) can be found in MoBa's instrument documentation (Jin, 2016).

Measures of gestational age and birth weight were collected from the Medical Birth Registry, which contains information on all births in Norway from 1967 and onwards (Irgens, 2000). Gestational age was centered on 40 weeks. Birth weight was included as SGA. This was a binary variable scored 1 for those who weighed less than 2 standard deviations below expected birth weight and zero otherwise, as defined by Marsál (Marsál et al., 1996).

### Statistical analyses

Horn's parallel analysis (Horn, 1965) was conducted as an initial test of how many latent factors to include. We proceeded by fitting several bifactor exploratory factor analysis (EFA) models to the data, and evaluated each latent factor on both goodness-of-fit and psychometric indices. Regarding psychometric indices, we emphasized the H-index (H > 0.70), which is a measure of how well the latent variable is defined by its indicators (Rodriguez, Reise, & Haviland, 2016). Other indices were also included for comprehensiveness (short descriptions in Table 1). These are discussed thoroughly elsewhere (Rodriguez et al., 2016). Next, we selected the best fitting model(s) and ran biometric EFA versions of these to investigate etiology and criterion validity. With respect to the biometric modeling, we distinguish between additive genetic- (A), non-additive/dominance genetic- (D), common environmental- (C) and unique environmental influences (E). The correlation structure of A and D among individuals was specified according to the additive and non-additive genetic correlations derived from the pedigree structure (described above). We defined C as an environmental component shared among individuals with the same mother, and E was defined as an environmental component unique to the individual. The common factor model assumes that the responses relating to an individual (***y***) can be described as
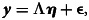
where 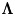 is the factor loading matrix, ***η*** a vector of common factors and 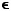 a vector of unique factors. In the biometric extension of the factor model we specified that the common and unique factors are a function of genetic and environmental components, e.g.:
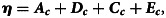

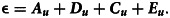

**Table 1.** Psychometric and goodness of fit indices for different factor solutions on the phenotypic exploratory factor analysis models with bifactor rotation

Index	Factors			
	P factor	F1	F2	F3
Bifactor model with three specific factors				
H	0.88	0.72	0.67	0.46
ECV	0.58			
Omega	0.64			
OmegaH	0.32	0.09	0.22	0.01
Factor determinacy	0.96	0.98	0.85	0.84
PUC	0.75			
AIC	295 739.4			
BIC	296 096.2			
RMSEA	0.069 (95% CI 0.065–0.072)			
CFI	0.97			
Bifactor model with two specific factors				
H	0.88	0.68	0.43	
ECV	0.67			
Omega	0.59			
OmegaH	0.37	0.22	0.00	
Factor determinacy	0.96	0.85	0.86	
PUC	0.76			
AIC	297 534.2			
BIC	297 832.7			
RMSEA	0.091 (95% CI 0.088–0.095)			
CFI	0.92			
Bifactor model with one specific factor				
H	0.86	0.69		
ECV	0.76			
Omega	0.58			
OmegaH	0.40	0.18		
Factor determinacy	0.93	0.85		
PUC	0.93			
AIC	302 362.6			
BIC	302 595.6			
RMSEA	0.1292 (95% CI 0.1263–0.1320)			
CFI	0.80			
One-factor model				
H	0.86			
ECV	1			
Omega	0.40			
OmegaH	0.40			
Factor determinacy	0.93			
PUC	1			
AIC	308 187.4			
BIC	308 347.6			
RMSEA	0.1534 (95% CI 0.1508–0.1559)			
CFI	0.66			

*Note*: only factor loadings >0.2 was included in the indices. F1 = first residual/specific latent factor; F2 = second residual/specific latent factor; F3 = third residual/specific latent factor. H = H index, a construct replicability index where high values reflect that the factor is well defined by its indicators. Threshold commonly used is >0.70; ECV = Explained common variance, a measure of strength of the general factor (the value indicates the proportion of the total variance in the indicators explained by the general rather than the specific latent factors); Omega = a measure of reliability, indicating the proportion of variance attributable to both the general and specific factors together; OmegaH = Omega hierarchical, a measure of reliability that estimates the proportion of variance attributable to the general factor only; Factor determinacy = an index of trustworthiness of the latent factor, where a high values indicates that the predicted factor scores correspond well with the corresponding factor. Threshold commonly used is >0.90; PUC = Percent uncontaminated correlations, an indicator of how many percent of all correlations among indicators attributable to the general factor (Hancock & Mueller, 2001; Rodriguez et al., 2016). AIC = Akaike's information criterium, a measure of a model's goodness of fit relative to other models, where parsimony is favored. The preferred model is the one with the lowest AIC value (Akaike, 1987); BIC = Bayesian information criterium, a relative goodness of fit index, similar to AIC, but with different penalizing of model complexity (Schwarz, 1978); RMSEA = Root mean square error of approximation, an absolute goodness of fit index, that assesses how far a hypothesized model is from a perfect model (Steiger, 1990). Threshold commonly used is <0.05; CFI = Comparative fit index (Bentler, 1990), an absolute goodness of fit index, similar to RMSEA but often used in exploratory contexts. Threshold commonly used is >0.95.

Given the current pedigree it is statistically difficult to distinguish C from D effects. We therefore ran ACE and ADE models separately. The six symptom clusters and five personality traits were first residualized on child sex. Full information maximum likelihood was used to fit the models, and the factor loadings matrix was rotated using the Jennrich–Bentler orthogonal bifactor rotation (Jennrich & Bentler, 2011) with the function bifactorT in the GPArotation package in R (Bernaards & Jennrich, 2005). Here, a single general factor is isolated that explains covariance between all symptom clusters and traits, in addition to residual latent factors that are uncorrelated with the general factor and explain residual covariance between clusters of variables not accounted for by the general factor (Jennrich & Bentler, 2011). Genetic and environmental sources of variance on the rotated common factors were estimated, along with genetic and environmental residual variance for each trait. We first estimated a full model, in which A, C/D and E influences were allowed both on the latent factors and the observed traits. We then tested fixing the C/D effects on the residuals of the observed traits to zero, while retaining them on the latent factors. The most restricted model was a model where C/D was fixed to zero both on latent factors and observed-trait residuals. The nested sub-models were compared to the full models using Akaike's information criterion (AIC; Akaike, 1987). As bifactor rotation solutions have been criticized for being unstable, we also simulated how stable the best fitting solution was in this dataset (eAppendix 2).

To investigate their associations with gestational age and birth weight, the general psychopathology factor as well as the residual latent factors was each regressed onto gestational age and SGA in a joint model including the best fitting biometric structure. Gestational age was allowed both a linear and quadratic association with the latent factors. The modeling procedures were conducted in R, using the svcmr package (code available at https://github.com/espenmei/svcmr).

## Results

### Model fitting

**Fig. 1.**
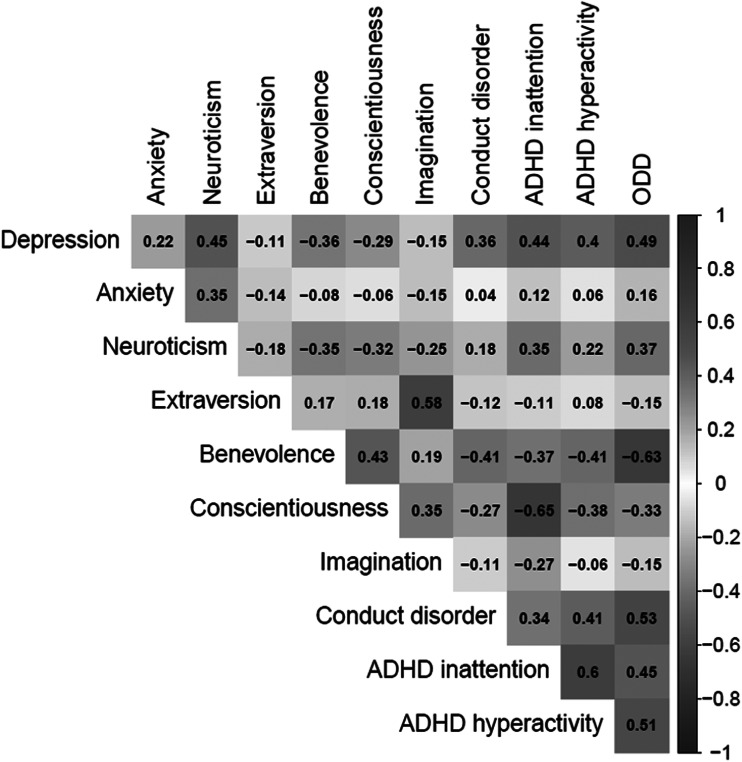
Correlations between included variables. *Note*: Gray cross indicates correlations not significant (*p* > 0.05).

**Fig. 2.**
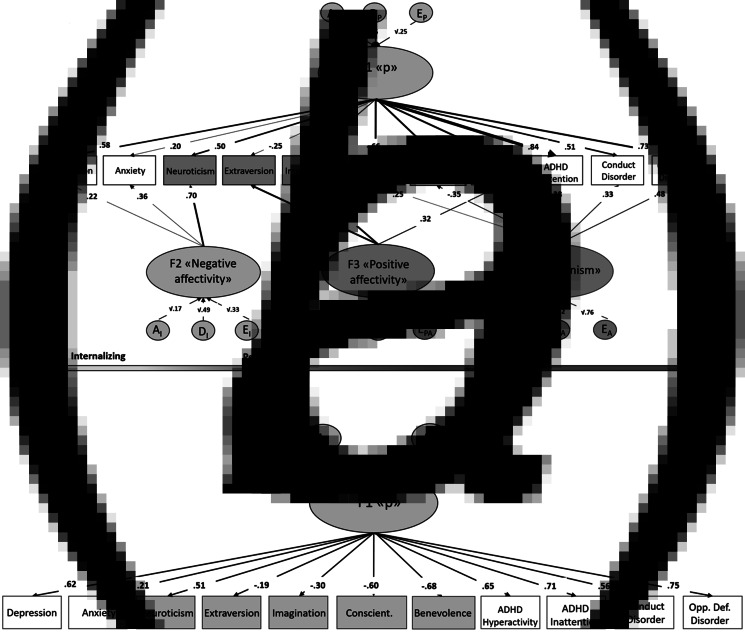
Best fitting EFA models. *Note*: Loadings below 0.20 are not shown in Fig. 2a. A = Broad-sense genetic influences (including both additive and non-additive/dominance effects); E = unique environmental influences and measurement error.

A correlation matrix of the traits is shown in Fig. 1. Horn's parallel test indicated three factors (online Supplementary Fig. S1). The four-factor model (Fig. 2a) had a superior fit according to the goodness-of-fit indices (Table 1) and was also highly stable in this dataset (eAppendix 2). However, only a one-factor model (Fig. 2b) satisfied psychometric criteria for interpretability (e.g. H > 0.7; Table 1).

Twin- and sibling correlations indicated that an ADE model would fit the data best (online Supplementary Fig. S2). This was confirmed by goodness-of-fit indices for both the four- and one-factor solution (Table 2). After bifactor rotation on the four-factor solution, a *p* factor (F1) was isolated. Similarly to the one-factor solution all symptom clusters and neuroticism had positive loadings and the other personality dimensions had negative loadings on this general factor (online Supplementary Table S1). Three residual latent factors also emerged: a negative affectivity factor (F2), with loadings on depression and anxiety symptoms and neuroticism, along with a positive affectivity factor (F3; loading onto extraversion, imagination and ADHD hyperactivity symptoms), and a less clear antagonism factor (F4) that resembled rule-breaking behavior (positive loadings on CD, oppositional defiant disorder and conscientiousness, and negative loadings on benevolence and inattention). Variance explained by the *p* factor and residual factors along with variance unique to the traits for both models are shown in online Supplementary Table S2. In the four-factor solution, the *p* factor explained most variance in ADHD inattention (70%; only 12% was unique to the trait), whereas for the one-factor model it was oppositional defiant disorder (57%; 43% unique to the trait).

**Table 2.** Model fit statistics from bifactor exploratory factor analyses

Model	−2LL	ep	AIC	Δdf	ΔLL	ΔAIC	*p*
Phenotypic factor solutions							
Four factor model	295 641	49	295 739	–	–	–	–
Three factor model	297 452	41	297 534	8	−1811	−1795	<0.00
Two factor model	302 298	32	302 362	9	−6657	−6623	<0.00
One factor model	308 143	22	308 187	10	−12 502	−12 448	<0.00
Four factor biometric models							
cACE sACE	−146 732	79	293 621	–	–	–	–
cACE sAE	−146 732	68	293 599	11	0	−22	1.00
cAE sAE	−146 733	64	293 593	15	1	−28	0.99
**cADE sADE**	**−146 688**	**79**	**293 535**	–	**–**	**–**	**–**
cADE sAE	−146 719	68	293 574	11	−31	39	<0.00
cAE sAE	−146 733	64	293 593	15	−45	58	<0.00
One factor biometric models							
cACE sACE	−152 949	46	305 990	–	–	–	–
cACE sAE	−152 949	35	305 968	11	0	22	1.00
cAE sAE	−152 949	34	305 966	1	0	24	1.00
**cADE sADE**	**−152 891**	**46**	**305 873**	–	**–**	**–**	**–**
cADE sAE	−152 949	35	305 968	11	58	−95	<0.00
cAE sAE	−152 949	34	305 966	1	58	−93	<0.00

Best fitting models are shown in bold. −2LL = two times the negative log likelihood – an estimate of how well the model fits the data; ep = number of estimated parameters included in the model; AIC = Akaike's information criterion – an indicator of how well the model fits the data that also penalizes complex models; df = degrees of freedom; ΔLL = the difference in log likelihood compared to the full model; *p* = probability value for rejecting the null hypothesis. cACE sACE = Additive genetic (A), shared environmental/shared mother effects (C) and unique environmental (E) effects on both common factors (c) and specific traits (s); cACE sAE = shared environmental/shared mother effects only on common factors and not specific traits; cADE sADE = Additive genetic (A), non-additive/dominance effects (D) and unique environmental (E) effects on both common factors (c) and specific traits (s); cADE sAE = non-additive/dominance effects only on common factors and not specific traits; cAE sAE = only additive genetic and unique environmental effects on both common factors and specific traits.

### Genetic and environmental contributions

The narrow-sense heritability of the *p* factor in the four-factor solution was 0.70, and dominance effects accounted for 0.05, giving a broad-sense heritability of 0.75. For the one-factor solution, only additive genetic influences contributed to the heritability (0.82). For the residual latent factors, the narrow-sense heritabilities were 0.17 for negative affectivity, 0.56 for positive affectivity, and 0.02 for antagonism, and dominance effects accounted for 0.49, 0.17, 0.22, giving broad-sense heritabilities of 0.67, 0.73, and 0.24, respectively. The rest of the variance in *p* and the residual latent factors was accounted for by unique environmental influences and measurement error. Residual broad-sense heritability spanned from 0.04 for neuroticism to 0.46 for anxiety (mean = 0.22; online Supplementary Table S3). Corresponding numbers for the one-factor solution were 0.11 ADHD inattention and 0.63 for imagination (mean = 0.37; online Supplementary Table S3). The rest of the variance was explained by unique environmental influences and measurement error.

As finding evidence for D over C in models of a wide range of psychopathology and personality traits was unexpected, and these traits were rated by mothers, it is possible the dominance effects reflect rater bias to some extent (Derks, Hudziak, & Boomsma, 2009). We therefore conducted sensitivity analyses on mono- and dizygotic twin pairs only to get an indication of whether sibling interaction or rater bias (Simonoff et al., 1998) could explain dominance effects (eAppendix 3). This was done using the method of adding an extra parameter that allows for feedback loops between siblings (Carey, 1986) on univariate biometric models of each trait separately as well as on a sum-score of the traits to resemble a *p* factor. We then compared goodness-of-fit between ADE models *v.* AE models with the added sibling feedback parameter. For most of the phenotypes, the AE + sibling feedback parameter fitted the data best.

### Associations between gestational age, SGA, *p* and residual latent factors

In the four-factor solution, children born SGA scored statistically significantly higher on negative affectivity compared to children not classified as SGA (*β* = 0.26, s.e. = 0.077, *p* = 0.001; online Supplementary Table S4). For *p* there was no difference in scores for SGA compared to non-SGA children (*p* = 0.838), nor for the two residual latent factors positive affectivity (*p* = 0.908) and antagonism (*p* = 0.160). In the one-factor solution, there was no association between *p* and SGA.

**Fig. 3.**
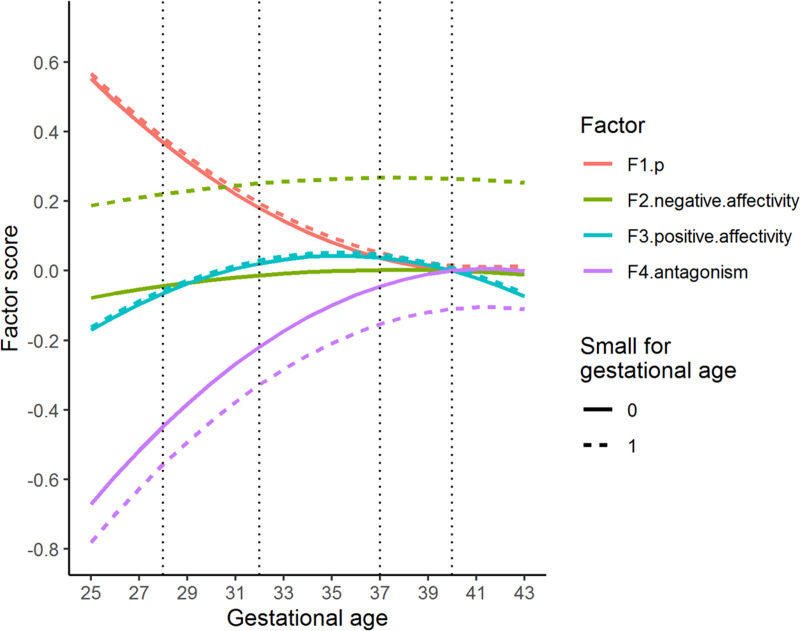
Predicted latent factor scores for different levels of gestational age. The figure shows the association between gestational age and *p*, as well as between gestational age and the residual latent factors (F2–F4), expressed as predicted factor scores for different levels of gestational age. Factors have mean = 0 and standard deviation of 1. The vertical lines index gestational age 40 (term), 37 (early term), 32 (very preterm) and 28 weeks (extremely preterm). Dotted lines are predicted factor scores for children born small for gestational age.

Low gestational age had a curvilinear, negative association with *p* that flattened as gestational age approached term in both the four-factor (*p* = 0.036) and one-factor solution (*p* = 0.002). For instance, children born in gestational week 28 were predicted to score ≈0.4 standard deviations (s.d.) higher on *p* compared to children born in gestational week 40 (Fig. 3 and online Supplementary Fig. S3). This pattern was very similar for both the one- and four-factor solution. For the four-factor solution, gestational age had a positive, curvilinear statistically significantly association with two of the three residual latent factors: positive affectivity (*p* = 0.046), and antagonism (*p* = 0.012). Children born in gestational week 28 were predicted a ≈0.42 s.d. lower score on antagonism compared to children born full term (week 40). There was no evidence of an interaction between gestational age and SGA on the factors.

## Discussion

The present study provides insight into the nature of psychopathology risk in middle childhood. A *p* factor could be recovered in eight-year-old children when personality was included in the structure, in line with what has been shown in adults (Kotov et al., 2017; Rosenström et al., 2018) and a spectrum model of psychopathology and personality (Widiger, 2011). According to our findings, the *p* factor in middle childhood is characterized by high scores on inattention, oppositional defiant behavior, and hyperactivity as well as low scores on conscientiousness and agreeableness (Fig. 2, online Supplementary Table S2). There are to our knowledge no other comprehensive, factorial studies on the joint structure of psychopathology and personality in middle childhood.

The *p* factor recovered in the present study fulfilled all criteria we defined for being a useful construct (capturing genetic and environmental risk, demonstrating psychometric properties for interpretability, and criterion validity). The *p* factor was also robust, as it was almost identical in the one- and four factor solution on loading pattern, heritability, and strength and direction of association with early putative risk factors.

Our findings contribute to the debate on what constitutes the core of the *p* factor in middle childhood. Some find it to be defined by internalizing aspects (Lahey et al., 2011; Waldman et al., 2016), some by externalizing and autism aspects (Allegrini et al., 2020; Martel et al., 2017; Moore et al., 2020; Neumann et al., 2016), and some by borderline personality traits which sit in between internalizing and externalizing spectra (Gluschkoff, Jokela, & Rosenström, 2021). Our study adds to the literature by linking established developmental risk factors and personality in an etiologically important age period to a model of the *p* factor. Here, the constellation of associations between normative personality traits and the *p* factor resembled that of normative personality and borderline personality disorder (Samuel & Widiger, 2008), as observed for adults (Rosenström et al., 2018). In the four- and one-factor models, the strongest and second strongest loadings, respectively, on the *p* factor was for ADHD inattention. ADHD has particularly strong etiological links to borderline personality disorder (Kuja-Halkola et al., 2021). Furthermore, our *p* factor was associated with early gestational age that is also a risk factor for ADHD inattention (Ask et al., 2018). Thus, we argue that the *p* factor may be a natural model for psychopathology that sits between traditional internalizing and externalizing spectra rather than being their re-expression.

It is worth commenting on why both the one- and four-factor solutions were included. Goodness-of-fit is often used when the main aim is to explore structure rather than to construct measurement instruments. In confirmatory modeling and when robust constructs or measures are of interest, the recommendation is to also include psychometric fit indices (Rodriguez et al., 2016) to ensure that included residual latent factors are interpretable and replicable. In the present study, none of the models performed well for all latent factors on both goodness-of-fit and psychometric indices. Thus, we used two models to show that the *p* factor was the same across the models and attained good performance on all indices. As we studied the correlation structure of 11 quite different psychopathology and personality traits, measured with different scales using an ESEM approach (Asparouhov & Muthén, 2009), we did not expect a clean psychometric measurement model. The ESEM strategy has been created precisely because such clean structures are often infeasible when underlying structures are of interest. Previous studies on the hierarchical structure of psychopathology usually need two residual latent factors in addition to *p* (e.g. Caspi et al., 2014; Lahey et al., 2011). When personality is added, no less residual factors should be needed. From a structural viewpoint, our multifactor model makes sense, and hopefully also appeal to some applied researchers that may take interest in the evidence for psychometrically valid scale constructs (as discussed for instance in Lahey, Moore, Kaczkurkin, & Zald, 2021).

In the four-factor solution, three residual latent factors in addition to *p* were necessary to explain covariance in the data. We interpreted these as a negative affectivity (F2), a positive affectivity (F3), and a less clear antagonism factor (F4). Contrary to the *p* factor, reliability estimates for these factors were sub-optimal (Hancock & Mueller, 2001; Rodriguez et al., 2016) and their interpretation is more imprecise. We therefore refrain from closer interpretation of their content.

The high broad-sense heritability of the *p* factor (75-82%) indicates that early etiology of psychiatric burden is driven by genetic risks. This is in line with previous studies on *p* in childhood and adolescent samples (Allegrini et al., 2020; Lahey et al., 2011; Waldman et al., 2016), although the influence of genes seems to be higher in our study. However, it is unusual to find evidence for non-additive genetic effects in etiological studies of the hierarchical structure of childhood psychopathology (e.g. Lahey et al., 2011). As we have included personality, the finding of D-effects makes sense as such effects have been found for personality traits and ADHD (Derks et al., 2009; Keller, Coventry, Heath, & Martin, 2005). Yet, as all our included traits had substantial D-effects, this finding may to some extent reflect rater contrast effects (Simonoff et al., 1998). This suspicion was supported by the sensitivity analyses (eAppendix 3), making this topic a feasible possibility for further study.

Understanding how personality relates to psychopathology can be valuable in clinical settings, since personality traits can be measured in young children before the onset of psychopathology. We have previously shown that when modeling the joint structure of psychopathology and personality in adults, all Big Five traits (except openness) load onto the *p* factor (Rosenström et al., 2018). The personality profile that best reflected *p* was a high score on neuroticism as well as low scores on conscientiousness and agreeableness. In this sample of eight-year-olds, the findings were similar, but instead low scores on conscientiousness and benevolence were most characteristic for p. Conscientiousness and benevolence even had higher loadings on *p* than many of the psychopathology traits. This finding extends those of previous studies where neuroticism is typically found to be most important, but is not surprising considering the centrality of poor self-regulation on developmental psychopathology (Nigg, 2017). Perhaps children presenting with behavior that resembles a profile of low conscientiousness and benevolence, along with high neuroticism should be followed more closely than children with a less risk-prone personality profile to prevent psychopathology. Our study cannot answer whether these traits predict risk for later psychopathology as the measurements were conducted at the same time-point, but personality has been shown to be relatively stable in childhood (Lamb, Chuang, Wessels, Broberg, & Hwang, 2002).

The *p* factor was negatively associated with gestational age, indicating that prematurely born children scored higher on general psychopathology risk. This finding, along with the high heritability and interpretability, supports the notion that *p* is a clinically relevant construct. We can only speculate on the mechanisms behind the association between *p* and gestational age. It is known that preterm birth compromises brain development (Davis et al., 2011) and is associated with smaller brain volume (Nosarti et al., 2002). It is biologically plausible that being born with an immature nervous system increases the risk of developing psychopathology (Nosarti et al., 2012). An immature nervous system may be more vulnerable to stressors, and it may be harder for parents to correctly interpret the cues from their preterm babies.

There are notable strengths in our study, such as the large sample size and the rich measurements of both psychopathology and personality traits. Some limitations also need to be acknowledged. First, all included traits were reported by mothers, rendering shared method bias possible (Podsakoff, MacKenzie, Lee, & Podsakoff, 2003). Our sensitivity analyses on a subset of the data indicated that rater bias was likely. We recommend that all researchers wanting to conduct family studies on MoBa data have this in mind, although further studies are required to conclude.

The anxiety measure had a low Cronbach's *α* value (0.48) and had a lower association with *p* than expected. This is possibly due to this instrument being constructed to measure a variety of anxiety disorders but could also be due to unreliability.

The recruitment rate in MoBa is low (41%; Magnus et al., 2006), and it has been found that women in MoBa differ from other childbearing women in Norway on several exposures and outcomes (Nilsen et al., 2009). It is possible that women with severe psychopathology symptoms did not participate, and that the children of these mothers differ from children of participating mothers on psychopathology or personality traits. However, the children, which are the focus of the present study, have not self-selected into the study, which may give less bias in this generation than in the parent generation. When recruitment rates are low, bias typically occur in estimates of prevalence, and not in estimates of associations (Nilsen et al., 2009).

Analysis indicated that gestational age was associated with both the *p* factor and residual factors. However, the standard errors of these estimates were high, indicating that these estimates are uncertain. Many variables are associated with SGA and gestational age, such as characteristics about the mothers (weight, medical history, smoking, etc.; McCowan & Horgan, 2009). Thus, the mechanism explaining the correlation between the *p* factor and gestational age require further study.

The bifactor rotation of the included traits provides just one of many possible factor structures of childhood psychopathology and personality. As this was an exploratory study, we did not test other solutions. The bifactor rotation is a common and recommended practice for studies on the hierarchical structure of psychopathology (Levin-Aspenson et al., 2020).

To sum up, this study extends previous findings on the nature and etiology of general psychopathology in middle childhood. Personality can be meaningfully placed within a joint structure of psychopathology risk in this age group. The psychometric properties, high heritability of p, and its associations with established developmental risk factors lend support to the usefulness of this construct.

## Supplementary material

For supplementary material accompanying this paper visit https://doi.org/10.1017/S0033291723000077. click here to view supplementary material
