# The *p* factor of psychopathology and personality in middle childhood: Genetic and gestational risk factors – Corrigendum

**DOI:** 10.1017/S0033291723000879

**Published:** 2023-07

**Authors:** Line C. Gjerde, Espen Moen Eilertsen, Tom A. McAdams, Rosa Cheesman, Terrie E. Moffitt, Avshalom Caspi, Thalia C. Eley, Espen Røysamb, Tom H. Rosenström, Eivind Ystrom

**Affiliations:** 1 Department of Mental Disorders, Norwegian Institute of Public Health, Oslo, Norway; 2 Promenta Research Center, University of Oslo, Oslo, Norway; 3 Centre for Fertility and Health, Norwegian Institute of Public Health, Oslo, Norway; 4 Social, Genetic & Developmental Psychiatry Centre, Institute of Psychiatry, Psychology & Neuroscience, King's College, London, UK; 5 Department of Psychology and Neuroscience, Duke University, Durham, USA; 6 Department of Child Development, Norwegian Institute of Public Health, Oslo, Norway; 7 Department of Psychology and Logopedics, Faculty of Medicine, University of Helsinki, Helsinki, Finland; 8 School of Pharmacy, University of Oslo, Oslo, Norway

The original publication of the article includes an error in Figure 1. In the published figure, non-significant correlations between the included psychopathology and personality traits are crossed out. However, all correlations are statistically significant at p < 0.001. The misrepresentation is due to a coding error, in which the correlation matrix was used to calculate p-values instead of the data frame. The error only affected the p values and not the correlation coefficients and was isolated to this particular descriptive analysis only. The main analyses and conclusions are completely unaffected. The corrected Figure 1 is shown below.

**Figure 1.**
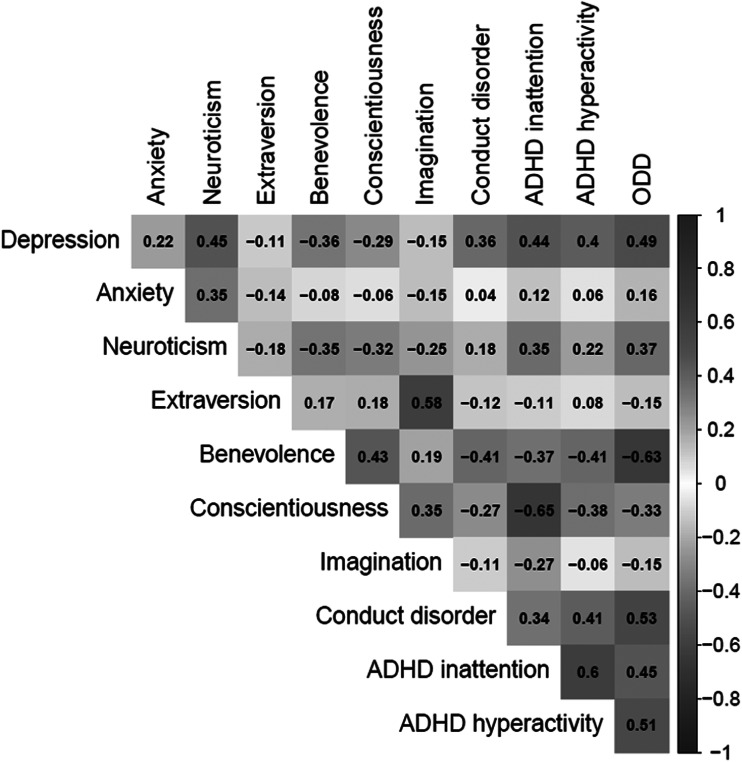
Correlations between included variables. Note: All correlations were statistically significant at p < 0.001.

The finding of non-statistically significant correlations was however commented on shortly with regard to the anxiety trait under the limitations section. The original sentence was:

“The anxiety measure had a low Cronbach's *α* value (0.48), was not significantly correlated with any of the other included traits and had a lower association with p than expected.”

This sentence should be replaced with:

“The anxiety measure had a low Cronbach's *α* value (0.48) and had a lower association with p than expected.”

The authors would like to thank Professor Emeritus Jean-Pierre Rolland at the University Paris Nanterre for asking the question that led us to discover the coding error.

