# Supplementary material for: The p factor of psychopathology and personality in middle childhood: genetic and gestational risk factors
Source: Psychol Med. 2023 Feb 10;53(9):4275–85. doi: 10.1017/S0033291723000077 (PMC10317823; doi:10.1017/S0033291723000077)
Supplement: Supplementary file 1 [file S0033291723000077sup001.docx]

Supplementary material

Figure S1


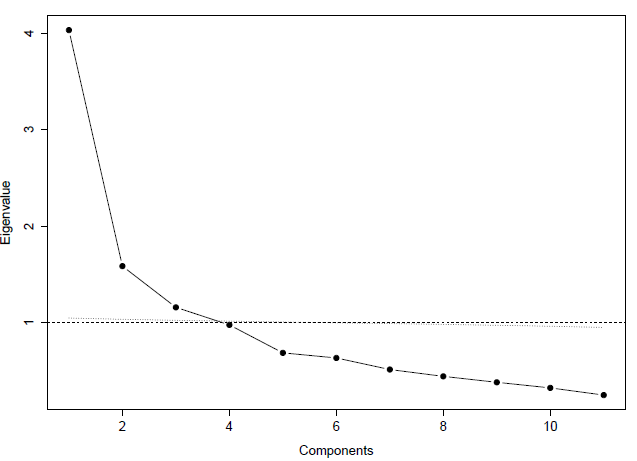


Simple scree plot showing the results from the parallel analysis.

Table S1

**Four factor solution with bifactor rotation One-factor solution**

F1 F2 F3 F4 F1

**Variable**

Depressive symptoms 0.58 0.22 0.10 0.11 0.62

Anxiety symptoms 0.20 0.36 -0.08 -0.01 0.21

Neuroticism 0.50 0.70 -0.03 0.01 0.51

Extraversion -0.25 -0.06 0.72 -0.07 -0.19

Benevolence/agreeableness -0.63 -0.02 0.01 -0.35 -0.68

Conscientiousness -0.66 0.04 0.08 0.25 -0.60

Imagination/Openness -0.38 -0.06 0.67 0.12 -0.30

Conduct disorder symptoms 0.51 -0.11 0.07 0.33 0.56

ADHD inattention symptoms 0.84 -0.08 0.14 -0.38 0.71

ADHD hyperactivity symptoms 0.65 -0.14 0.32 0.03 0.65

Oppositional defiant disorder symptoms 0.73 0.01 0.09 0.48 0.75

Factor loadings >.20 are shaded.

Table S2

Proportions of phenotypic variance explained by p and residual latent factors and unique to the traits (residuals) in the best fitting EFA model

**Four-factor solution with bifactor rotation One-factor solution**

F1 F2 F3 F4 Residual F1 Residual

DEP 0.34 0.05 0.01 0.01 0.59 0.38 0.62

ANX 0.04 0.13 0.01 0 0.82 0.05 0.95

NEU 0.25 0.49 0 0 0.26 0.26 0.74

EXT 0.06 0 0.52 0 0.41 0.04 0.96

BEN 0.39 0 0 0.12 0.48 0.46 0.54

CON 0.43 0 0.01 0.06 0.49 0.36 0.64

IMA 0.14 0 0.44 0.01 0.40 0.09 0.91

CD 0.26 0.01 0 0.11 0.62 0.31 0.69

ADHD_IN 0.70 0.01 0.02 0.15 0.12 0.50 0.50

ADHD_HY 0.42 0.02 0.10 0 0.46 0.42 0.58

ODD 0.53 0 0.01 0.23 0.23 0.57 0.43

F1 = p factor; F2 = negative affectivity factor; F3 = positive affectivity factor; F4 = “antagonism” factor. DEP = depressive symptoms; ANX = Anxiety symptoms; NEU = Neuroticism; EXT = Extraversion; BEN = Benevolence/Agreeableness; CON = Conscientiousness; IMA = Imagination/Openness; CD = Conduct disorder symptoms; ADHD_IN = ADHD inattention symptoms; ADHD_HY = ADHD hyperactivity symptoms; ODD = Oppositional defiant disorder symptoms.

Table S3

Proportions of total variance in traits split into genetic and unique environmental contributions shared with p and the residual latent factors and genetic and unique environmental contributions to residuals

**Four-factor solution**

Shared H^2^ Shared e^2^ Residual H^2^ Residual e^2^ Total H^2^ Total e^2^

DEP 0.32 0.09 0.36 0.23 0.68 0.32

ANX 0.13 0.05 0.46 0.36 0.59 0.41

NEU 0.55 0.19 0.04 0.22 0.59 0.41

EXT 0.44 0.16 0.16 0.24 0.60 0.40

BEN 0.38 0.14 0.20 0.28 0.58 0.42

CON 0.31 0.20 0.19 0.30 0.50 0.50

IMA 0.44 0.16 0.23 0.17 0.67 0.33

CD 0.27 0.11 0.40 0.21 0.68 0.32

ADHD_IN 0.49 0.39 0.07 0.05 0.56 0.44

ADHD_HY 0.39 0.15 0.17 0.29 0.56 0.44

ODD 0.54 0.23 0.13 0.10 0.68 0.32

**One-factor solution**

Shared H^2^ Shared e^2^ Residual H^2^ Residual e^2^ Total H^2^ Total e^2^

DEP 0.31 0.07 0.37 0.25 0.68 0.32

ANX 0.04 0.01 0.52 0.43 0.56 0.44

NEU 0.21 0.05 0.37 0.37 0.58 0.42

EXT 0.03 0.01 0.55 0.41 0.58 0.42

BEN 0.38 0.09 0.25 0.28 0.63 0.37

CON 0.29 0.07 0.23 0.41 0.52 0.48

IMA 0.07 0.02 0.63 0.28 0.70 0.30

CD 0.25 0.06 0.48 0.21 0.73 0.27

ADHD_IN 0.41 0.09 0.11 0.39 0.52 0.48

ADHD_HY 0.34 0.08 0.28 0.30 0.62 0.38

ODD 0.47 0.11 0.30 0.13 0.78 0.23

DEP = depressive symptoms; ANX = Anxiety symptoms; NEU = Neuroticism; EXT = Extraversion; BEN = Benevolence/Agreeableness; CON = Conscientiousness; IMA = Imagination/Openness; CD = Conduct disorder symptoms; ADHD_IN = ADHD inattention symptoms; ADHD_HY = ADHD hyperactivity symptoms; ODD = Oppositional defiant disorder symptoms. H^2^ = broad-sense heritability/genetic contribution; e^2^ = unique environmental contribution.

Table S4

Associations between gestational age, being small for gestational age, p, and residual latent factors

|  | p factor | | | Negative affectivity | | | Positive affectivity | | | Antagonism | | |
| --- | --- | --- | --- | --- | --- | --- | --- | --- | --- | --- | --- | --- |
|  | B | SE | p value | B | SE | p value | B | SE | p value | B | SE | p value |
| Four-factor solution |  |  |  |  |  |  |  |  |  |  |  |  |
| Gestational weeks | -0.0060 | 0.008 | 0.438 | -0.0023 | 0.008 | 0.773 | **-0.0187** | **0.008** | **0.037** | 0.0073 | 0.008 | 0.362 |
| Gestational weeks squared | **0.0021** | **0.001** | **0.036** | -0.0005 | 0.001 | 0.617 | **-0.0020** | **0.001** | **0.046** | **-0.0025** | **0.001** | **0.012** |
| Small for gestational age | 0.0149 | 0.073 | 0.838 | **0.2648** | **0.077** | **0.001** | 0.0090 | 0.078 | 0.908 | -0.1096 | 0.078 | 0.160 |
|  |  |  |  |  |  |  |  |  |  |  |  |  |
| One-factor solution |  |  |  |  |  |  |  |  |  |  |  |  |
| Gestational weeks | 0.0026 | 0.008 | 0.745 |  |  |  |  |  |  |  |  |  |
| Gestational weeks squared | **0.0031** | **0.001** | **0.002** |  |  |  |  |  |  |  |  |  |
| Small for gestational age | -0.044 | 0.066 | 0.505 |  |  |  |  |  |  |  |  |  |

B = Standardized regression coefficients. Statistically significant estimates are shown in bold. Gestational age is centered on full term (40 weeks), and hence the estimates are small.

Figure S2


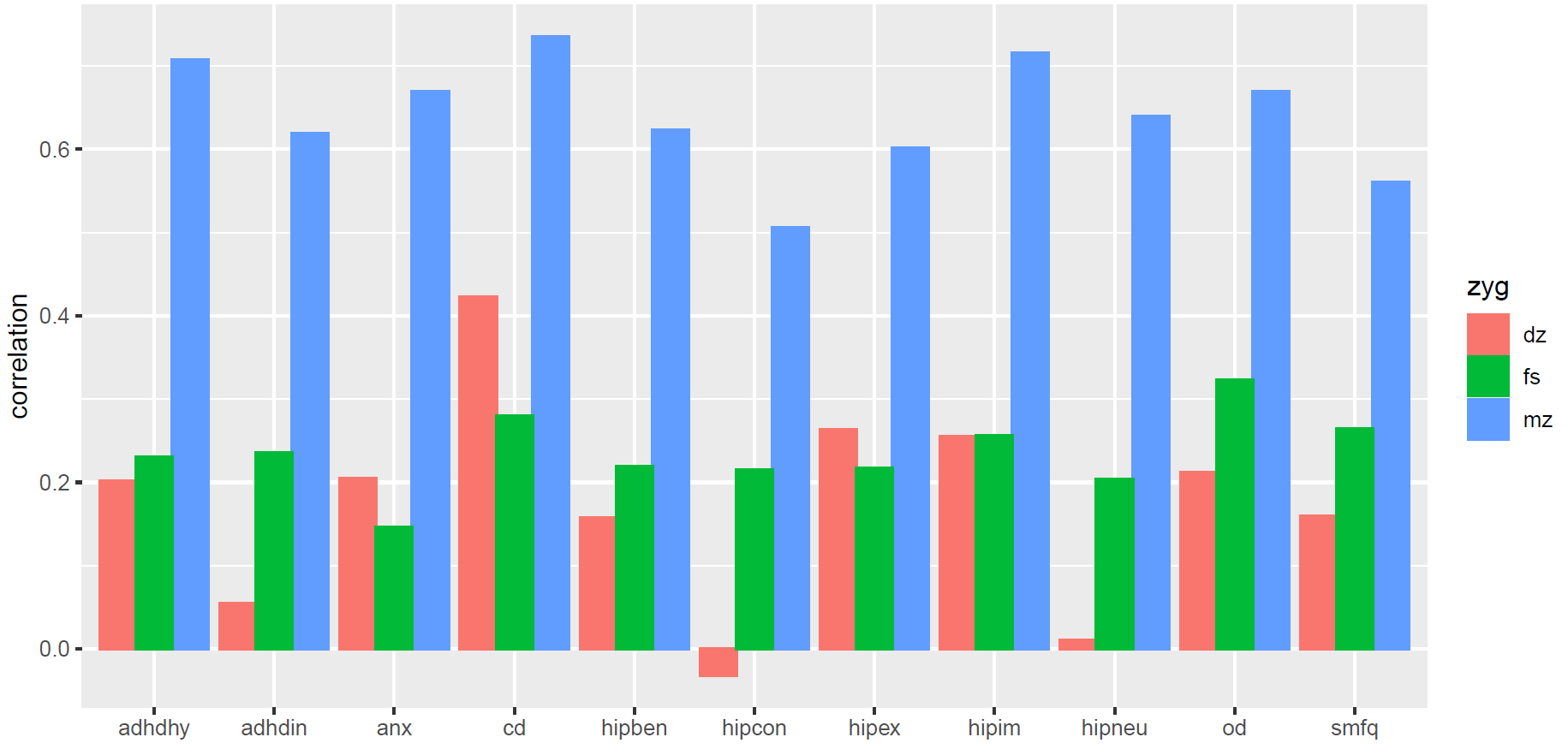


Phenotypic cross-siblin within-trait correlations for the monozygotic twin (mz), dizygotic twin (dz), and full sibling (fs) pairs for all the included psychopathology and personality scales. It should be noted that there are very few individuals in the mz and dz groups compared to the fs group. The correlations within the mz and dz group must therefore be interpreted with care. Adhdhy = AdHD hyperactivity; adhdin = ADHD inattention; anx = anxiety; cd = conduct disorder; hipben = benevolence; hipcon = conscientiousness; hipex = extraversion; hipim = imagination; hipneu = neuroticism; od = oppositional defiant disorder; smfq = depression.

Figure S3

Predicted latent factor scores for different levels of gestational age.


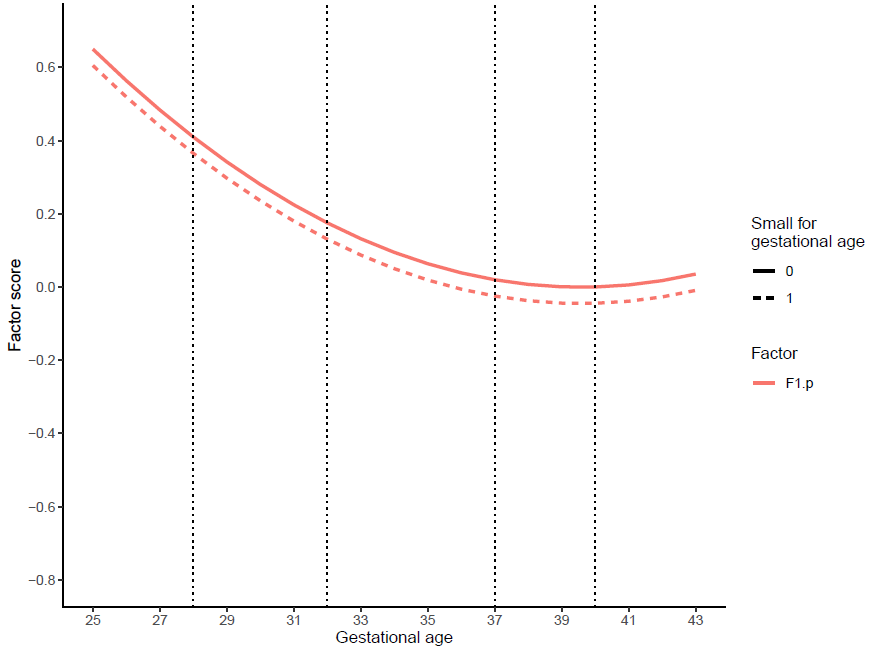


The figure shows the association between gestational age and p expressed as predicted factor scores for different levels of gestational age. Factors have mean = 0 and standard deviation of 1. The vertical lines index gestational age 40 (term), 37 (early term), 32 (very preterm) and 28 weeks (extremely preterm). Dotted lines are predicted factor scores for children born small for gestational age.

eAppendix 1

For the offspring of the MoBa participants, we determined genetic relatedness from kinship information in MoBa. For same sex twins, maternal reports on zygosity were obtained using questionnaire items administered by phone or mail, and a sub-group of the same-sex offspring twins was also genotyped. A logistic regression model, regressing genotype classifications on the questionnaire items was fit to the twin pairs having both measurements. The fitted model was then used to classify the twin pairs that had not been genotyped, based on the questionnaire responses. The discrepancy between classification by questionnaire and genotyping had an expected misclassification rate of <4% in our sample.

eAppendix 2

**SUPPLEMENTARY ROBUSTNESS ANALYSIS: The p factor of psychopathology and personality in middle childhood: Genetic and gestational risk factors.**

Mansolf and Reise (2016) provided a single simulation condition, based on which they argued that analytic exploratory bifactor rotations by Jennrich and Bentler (2011) yield unstable factor loadings. Such claims cannot be generalized from a single case, however, and the matter should be investigated on a case-by-case basis until very extensive simulations have been published. Therefore, we provide this supplementary simulation analysis to demonstrate that our bifactor loadings were stable to all practical purposes.

By unstable factor loadings, Mansolf and Reise (2016) meant the following. Exploratory factor loadings are not uniquely defined by the statistical factor analysis model but this rotational indeterminacy can be fixed via some of heuristic rotation criterion (e.g., Lawley & Maxwell, 1971). The exploratory bifactor rotation is determined as a minimum of a certain objective function of the factor loadings, attaining of which sets the factor loading values. Mansolf and Reise noticed that this objective criterion can have local minima, evidence by different solutions being obtained from different (arbitrarily chosen) starting rotation matrices. In this sense, an individual set of factor loadings can be unstable. The practical implication is that a different investigator could end up with different values with either just slightly different data or different starting values. However, little is still known about how frequent phenomenon such instability is in practice for some specific data. Luckily, for a specific dataset, the issue is easy to explore. We did it as follows.

We generated uniformly distributed square orthonormal random matrices (uniformly distributed on the Stiefel manifold of orthonormal 4-frames) using the function “rustiefel” from the R package “rstiefel”, version 1.0.1. A generated random matrix A was rejected when det(A) = -1 and accepted when it was 1 (these are only possible values), until we had 1000 independent random rotation matrices. Such matrices are elements of special orthogonal group (i.e., “rotations”, while “reflections” were rejected). Each of the 1000 random rotation matrices were used as new starting (rotation) values for the analytic bifactor rotation, thereby producing 1000 alternative bifactor solutions.

For all the 1000 random initial values, our data led to the exact same objective criterion value of 0.6582, up to four decimal place’s numeric precision. Thus, no local minima appeared. When multiplying individual loadings with their signs to resolve the inherent sign-indeterminacy of underlying factors, there was practically no variance in the individual factor loadings (Supplementary Figure S2). Thus, no multiple distinct global minima appeared either. That is, our exploratory factor loadings did not seem unstable at all in this simulation test of solution stability.


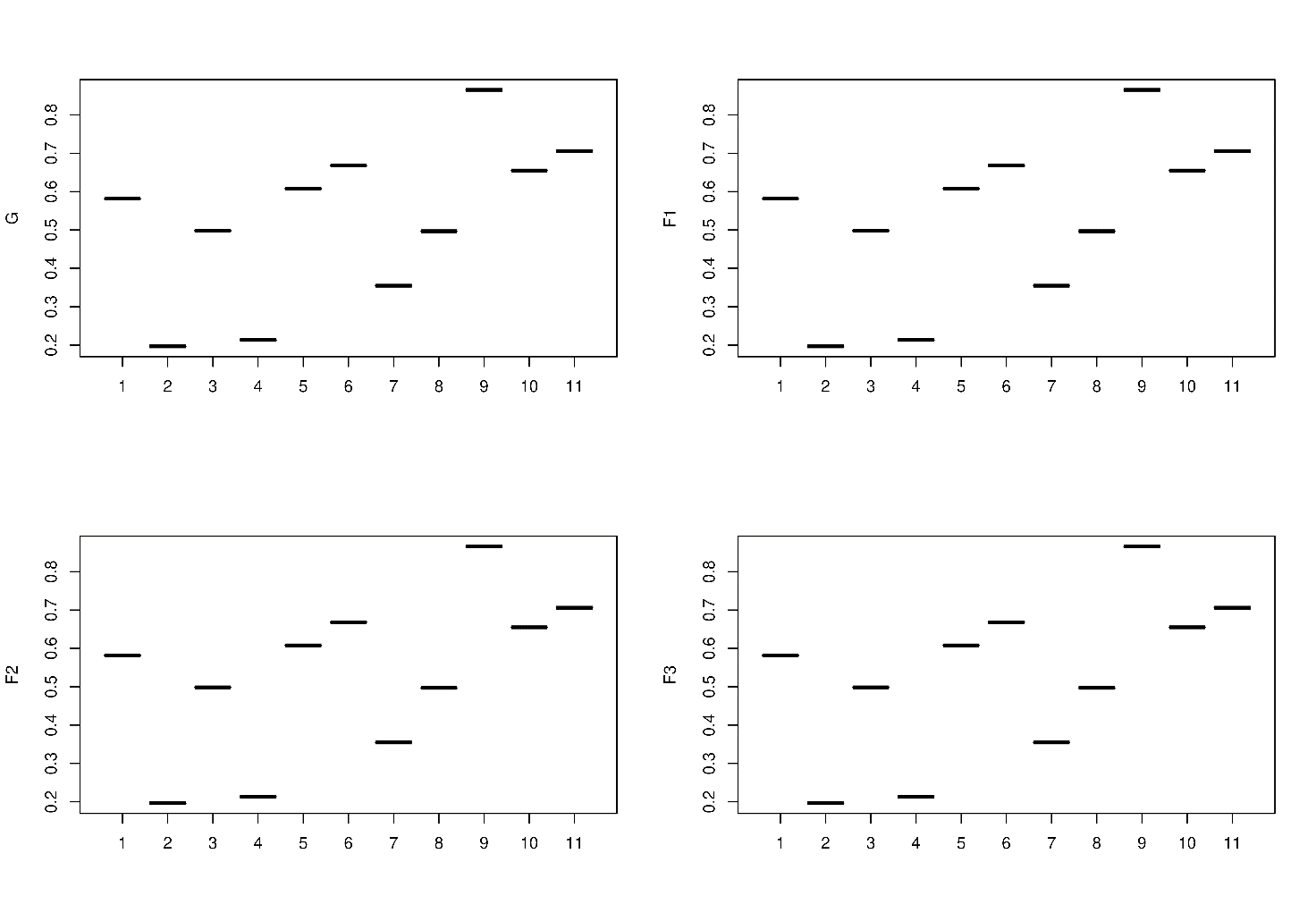


Supplementary Figure S4. Boxplots of absolute values for all bifactor loadings across 1000 bifactor rotation solutions from different random starting values (i.e., from 1000 different random rotation matrices). Y-axis gives the factor number, with “G” denoting the general factor and “F” specific factors. As there is practically no variance in single factor loadings compared to the cross-loading differences, the boxes of the “boxplot” show up as mere lines, meaning that the factor loadings were highly stable.

**References**

Jennrich, R. I., & Bentler, P. M. (2011). Exploratory bi-factor analysis. *Psychometrika*, *76*(4), 537–549. https://doi.org/10.1007/s11336-011-9218-4

Lawley, D. N., & Maxwell, A. E. (1971). *Factor Analysis as a Statistical Method.* (2nd ed.). Butterworths & Co.

Mansolf, M., & Reise, S. P. (2016). Exploratory Bifactor Analysis: The Schmid-Leiman Orthogonalization and Jennrich-Bentler Analytic Rotations. *Multivariate Behavioral Research*, *51*(5), 698–717. https://doi.org/10.1080/00273171.2016.1215898

eAppendix 3

**SUPPLEMENTARY ANALYSIS: Comparison of models indicating genetic dominance versus sibling interaction/rater bias.**

One of the reviewers suggested that we conducted additional analyses to get an indication of whether the low dizygotic- compared to monozygotic twin correlations were due to genetic dominance effects or sibling interaction/rater contrast effects. As we are not aware of examples of multivariate models based on complex pedigree structure data applying the method described in the paper by Carey (Carey, 1986), we ran a set of univariate models for each of the 11 scales included in the exploratory factor analysis models using only mono- and dizygotic twin pairs. We also summed the 11 scales into a sum-score to approximate the p factor. For each phenotype (scale) we ran an ADE model and an AE + sibling interaction/rater bias path. We then compared the goodness of fit in terms of AIC of each of these sets to get an indication of whether true genetic dominance effects or sibling interaction/rater bias effects was most likely. As can be seen from the figure below, for most phenotypes, the AE + sibling interaction/rater bias model fitted the data best. This finding indicates that the genetic dominance effects may be spurious.

Figure S5


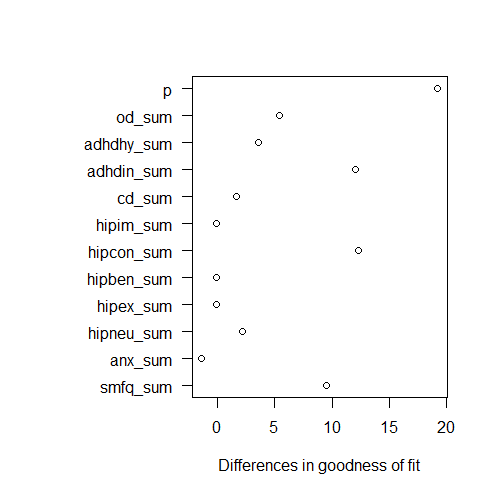


The Y axis indexes the AIC value of the ADE model minus the AIC value of the AE + sibling interaction/rater bias effects. A positive number means that the AE + sibling interaction/rater bias effects fitted the data best. P = p factor; od_sum = sumscore of items for oppositional defiant disorder; Adhdhy_sum = AdHD hyperactivity; adhdin_sum = ADHD inattention; cd_sum = conduct disorder; hipim_sum = imagination; hipcon_sum = conscientiousness; hipben_sum = benevolence; hipex = extraversion; hipneu_sum = neuroticism; anx_sum = anxiety; smfq_sum = depression.

**References**

Carey, G. (1986). Sibling imitation and contrast effects. *Behavior Genetics, 16*(3), 319-341.
